# Influence of Heart Function on the Assessment of MASLD Using Indocyanine Green Clearance

**DOI:** 10.1007/s11695-026-08560-1

**Published:** 2026-02-28

**Authors:** Jure Salobir, Branislava Ranković, Tadeja Pintar Kaliterna

**Affiliations:** 1 Faculty of Medicine, University of Ljubljana, Ljubljana, Slovenia; 2 Department of Abdominal Surgery, Ljubljana University Medical Centre, Ljubljana, Slovenia

## Abstract

**Introduction:**

The incidence of MASLD is rapidly increasing while the accuracy of noninvasive tests for MASLD remains limited. Indocyanine green clearance is a dynamic liver function test established for the evaluation of liver disease but is limited by the major influence of systemic circulation. We aimed to assess the role of Indocyanine green clearance in the evaluation of MASLD, together with cardiac function measurements.

**Methods:**

We conducted a prospective controlled study of 77 patients who underwent metabolic and bariatric surgery (MBS) and 25 controls who underwent elective cholecystectomy. Indocyanine green clearance was measured and interpreted as plasma disappearance rate (PDR) and residual dose at 15 min (R15). Wedge liver biopsies were obtained during MBS and assessed using the Kleiner classification. In the observed group cardiac output and cardiac index were measured and used to evaluate the influence of cardiac function on liver function monitoring.

**Results:**

Indocyanine green clearance was significantly lower in the observed patients compared to controls (*p* < 0.05). PDR was significantly associated with a pathological histological score (*p* = 0.03). Cardiac output and cardiac index were independently associated with R15 (*p* < 0.01). PDR adjusted for cardiac output and cardiac index significantly corelated with liver fibrosis, revealing a relationship not evident in unadjusted measurements.

**Conclusions:**

Indocyanine green clearance reflects MASLD severity in MBS patients. Its diagnostic value is further enhanced by adjusting for cardiac function. This novel combined approach may improve noninvasive assessment of MASLD and support treatment individualization in patients preparing for MBS.

## Key points

ICG clearance is impaired in patients with MASLD.

Cardiac function influences liver function assessment using ICG clearance.

Adjusting PDR for cardiac function improves correlation with liver fibrosis.

## Introduction

Metabolic and bariatric surgery (MBS) is a crucial treatment option for obesity and its complications. Among these, metabolic dysfunction-associated steatotic liver disease (MASLD) is increasingly recognized as an important treatment target. MBS can resolve metabolic dysfunction-associated steatohepatitis (MASH) and improve hepatic fibrosis depending on factors such as disease severity and the chosen procedure [1–3]. An accurate evaluation of MASLD is thus needed to identify MASLD, guide treatment, and monitor response.

The reference standard in the diagnosis of MASLD is liver biopsy which carries prohibitive logistic, cost and risk concerns for wider clinical use. Noninvasive tests (NIT) are thus needed to aid in diagnosis and reduce the need for biopsy.

Indocyanine green (ICG) clearance is the most common dynamic measurement of liver function and is widely utilized in liver surgery and intensive care medicine [4–10]. ICG is a water-soluble tricarbocyanine dye that is exclusively cleared by hepatocytes in its unaltered form [11]. Under normal conditions, ICG clearance is dependent on the rate-limiting effect of hepatic blood flow; with the progression of liver disease, the effect of cellular uptake and biliary excretion becomes more pronounced [9, 11]. The intravenous ICG concentration can be measured using pulse dye densitometry with a transcutaneous sensor. Thus, a dilution curve is measured to provide two measurements: ICG plasma disappearance rate (ICG-PDR) and ICG retention rate (ICG-R15) [9].

Due to the great influence of hepatic blood flow on ICG clearance, it has been suggested that cardiac output (CO) should be considered in the interpretation of clinical results [12]. However, we are unaware of any models that would incorporate CO in the interpretation of ICG clearance results.

This prospective controlled study tested three hypotheses: [1] ICG clearance is reduced in patients with MASLD undergoing MBS compared with control patients with BMI < 30; [2] ICG clearance parameters (ICG-PDR and ICG-R15) correlate with histological severity of MASLD as assessed by liver biopsy; and [3] Adjusting ICG clearance for cardiac function parameters (cardiac output and cardiac index) enhances its correlation with histological features. We expected that incorporating cardiac function into the interpretation of ICG clearance would improve its diagnostic accuracy and clinical utility as a noninvasive marker of liver function in patients with MASLD.

## Materials and Methods

### Study Design

We conducted a prospective controlled study between January 2020 and December 2023 to evaluate prospectively collected clinical, hemodynamic, and histological data. The study group included patients who underwent elective MBS. ICG clearance was measured and compared with the histological assessment of liver biopsies obtained intraoperatively. In a subgroup of patients, cardiac function was measured at the time of ICG clearance measurement. The control group included otherwise healthy patients scheduled for elective cholecystectomy, in whom ICG clearance was measured preoperatively. Patient data were anonymized and stored in a secure database. Written consent was obtained from each patient before their enrolment. All procedures performed in this study were in accordance with the ethical standards of the National Ethics Committee and the 1964 Helsinki Declaration and its later amendments.

### Patients

The study group included patients scheduled for elective MBS procedures. The exclusion criteria were a history of liver disease other than MASLD, current infection, a history of cancer (< 5 years), and alcohol intake exceeding 20 g/day (women) or 30 g/day (men). Patient assessment included medical history, physical examination, and anthropometric measurements. Regardless of study enrolment, all patients received standard clinical management. All surgeries were performed laparoscopically by a single surgeon.

The control group included patients scheduled for elective cholecystectomy due to gallstones or gallbladder polyps. The exclusion criteria were BMI ≥ 30, a history of liver disease, other comorbidities requiring treatment, a current infection, clinical or laboratory signs of biliary obstruction at the time of surgery, a history of cancer, gallbladder cancer found on histologic evaluation, and alcohol intake exceeding 20 g/day (women) or 30 g/day (men). Patient assessment included medical history, physical examination, and anthropometric measurements. Laparoscopic cholecystectomy was performed in all cases.

Additionally, due to the iodine content in ICG, patients with a known allergy to iodine, hyperthyroidism, thyroid tumors, or a known allergy to ICG were excluded.

Informed consent was obtained from all the patients included in this study.

### ICG Clearance Measurement

ICG clearance was measured in the study and control groups a day prior to or on the day of surgery. To minimize the variability of systemic circulation, all measurements were made in a fasting state in a supine position. A finger sensor connected to the LiMON device (Pulsion Medical Systems, Munich, Germany) was used for the measurements. ICG (Indocyanine Green, Verdy, Diagnostic Green GmbH, Aschheim-Dornach, Germany) was administered via a peripheral vein at a dose of 0.25 mg/kg. The results were recorded as ICG-PDR (%/min) and ICG-R15 (%).

### Heart Function Measurement

In a subset of the study group, heart function measurements were performed at the time of ICG clearance measurement. Cardiac function was non-invasively monitored using a finger-mounted ClearSight system (Edwards Lifesciences Corporation, Irvine, California, USA). A system that uses photoplethysmography to measure arterial volume and determine stroke volume, heart rate, CO, and cardiac index (CI), which were recorded at the time of ICG bolus injection.

### Liver Biopsy

In the study group, a wedge biopsy of the left lateral liver edge was obtained during MBS using a harmonic scalpel. The sample was fixed in formalin and sent for histological examination performed by a single pathologist. Evaluation was based on the Kleiner score with grading for steatosis (0–3), lobular inflammation (0–3) and hepatocyte ballooning (0–2) which gave a sum for a composite activity score ranging from 0 to 8, here referred to as the MASLD Activity Score (MAS), and a separate score for the evaluation of fibrosis ranging from 0 to 4 [13]. The term MAS is used for what was formerly known as NAFLD Activity Score (NAS) to ensure consistency with contemporary nomenclature, while maintaining the original histological scoring system and thresholds to preserve methodological validity and comparability with prior literature. Accordingly, patients with MAS/NAS < 3 were considered “not MASH”, those with MAS/NAS 3–4 were categorized as borderline MASH, and patients with MAS/NAS ≥ 5 were classified as having histological MASH.

### Statistical Analysis

Statistical analyses were performed in R (version 4.4.3) using the packages *tidyverse* for data manipulation and visualization, *janitor* for data cleaning, *readxl* for data import, *ggstatsplot* and *sjPlot* for statistical visualizations, *robustlmm* and *robustbase* for robust model estimation, *performance* for model diagnostics, *naniar* for missing data exploration, and *mice* for multiple imputation [14–22]. Continuous variables were summarized as means with standard deviations, medians with interquartile ranges, and ranges, categorical variables as absolute and relative frequencies. Outliers were detected using the interquartile range method. Missing data patterns were assessed, and multiple imputation with Predictive Mean Matching was applied to generate eight imputed datasets. Group comparisons were performed using the Mann–Whitney U test or the Kruskal–Wallis one-way ANOVA, followed by Holm-adjusted post hoc tests where applicable. Associations between variables were evaluated with Spearman’s rank correlation. Simple and multiple linear regression models were fitted, with model assumptions assessed by checking linearity, homoscedasticity, normality of residuals, and multicollinearity using variance inflation factors. Adjusted parameters were calculated by normalizing variables to relevant physiological measures, and regression analyses were also applied to non-invasive clinical scores. Statistical significance was set at a two-tailed p-value < 0.05.

## Results

### Patients Characteristics – Study Group

A total of 77 patients were enrolled in the study group. The mean age was 48,9 years (SD = 9.4, range 26–77 years). 20 patients (26.0%) were male and 57 (74.0%) were female. The mean body mass index (BMI) was 43.9 kg/m^2^ (SD = 8.1; range 27.5–69.4 kg/m^2^). 56 (73.7%) patients had metabolic syndrome (MS), 75 (97.4%) had associated comorbidities, including arterial hypertension (19.1%), T2DM (13.7%), hyperlipidemia (12.7%), obstructive sleep apnea (10.8%), and other cardiovascular (6.9%) and respiratory (5.9%) diseases. According to the American Society of Anesthesiologists Physical Status Classification System (ASA) 3 patients (3.9%) were ASA 1, 36 (46.8%) ASA 2, 37 (48.1%) ASA 3, and 4 (1.3%) ASA 4. One Anastomosis Gastric Bypass (OAGB) was performed in 60 patients (77.9%), sleeve gastrectomy was performed in 7 patients (9.1%), Roux-en-Y Gastric Bypass (RYGB) in 2 patients (2.6%), Single Anastomosis Sleeve Ileal Bypass (SASI) in 2 patients (2.6%), conversion of sleeve gastrectomy into OAGB in 4 patients (5.2%), and conversion of sleeve gastrectomy into RYGB in 2 patients (2.6%).

### Patient Characteristics – Control Group

25 patients were enrolled in the control group. The mean age was 43.3 years (SD = 14.3, range 16–46 years. The mean BMI was 25.2 kg/m^2^ (SD = 3.5, range 18.8–29.8 kg/m^2^).

### Liver Biopsy

Liver biopsies were performed in all patients in the study group. Table 1 shows the results of the histological evaluation. According to MAS, 29 patients (37.7%) were at a low risk of MASH, 29 (37.7%) were borderline, and 19 (24.7%) exhibited scores correlating with MASH. Overall, the mean MAS was 3.2 (SD 2, range 0–7).

**Table 1:** The results of histologic evaluation according to Kleiner [13]. 29 patients had MAS of < 3, 29 had a MAS of 3–4 and 19 had a MAS ≥ 5

Variable	Mean (SD)	Min < Med < Max	IQR (CV)	0	1	2	3	4
Steatosis	1.5 (1.0)	0 < 1 < 3	1 (0.6)	9 (11.7%)	35 (45.5%)	16 (20.8%)	17 (22.1%)	N/A
Lobular inflammation	0.9 (0.7)	0 < 1 < 2	1 (0.8)	22 (28.6%)	43 (55.8%)	12 (15.6%)	0	N/A
Hepatocyte ballooning	0.8 (0.6)	0 < 1 < 2	1 (0.8)	25 (32.5%)	43 (55.8%)	9 (11.7%)	N/A	N/A
Fibrosis	0.8 (0.7)	0 < 1 < 4	1 (0.9)	27 (35.1%)	42 (54.5%)	7 (9.1%)	0	1 (1.3%)

### ICG Clearance

ICG clearance was successfully measured in all enrolled patients without any side effects. In the study group the mean ICG-PDR was 23.5% (SD = 7.3, range 8.3–43.4, coefficient of variation (CV) = 31%) and the mean ICG-R15 was 4.8 (SD = 5, range 0.7–24.8, CV = 104.2%). In the control group the mean ICG-PDR was 27.2% (SD = 7, range 17.4–44.4, CV = 25,7%) and the mean R15 2.5 (SD = 1.9, range 0.1–2.4, CV = 76,0%).

### ICG Clearance Comparison Between the Study and Control Group

**Fig. 1:**
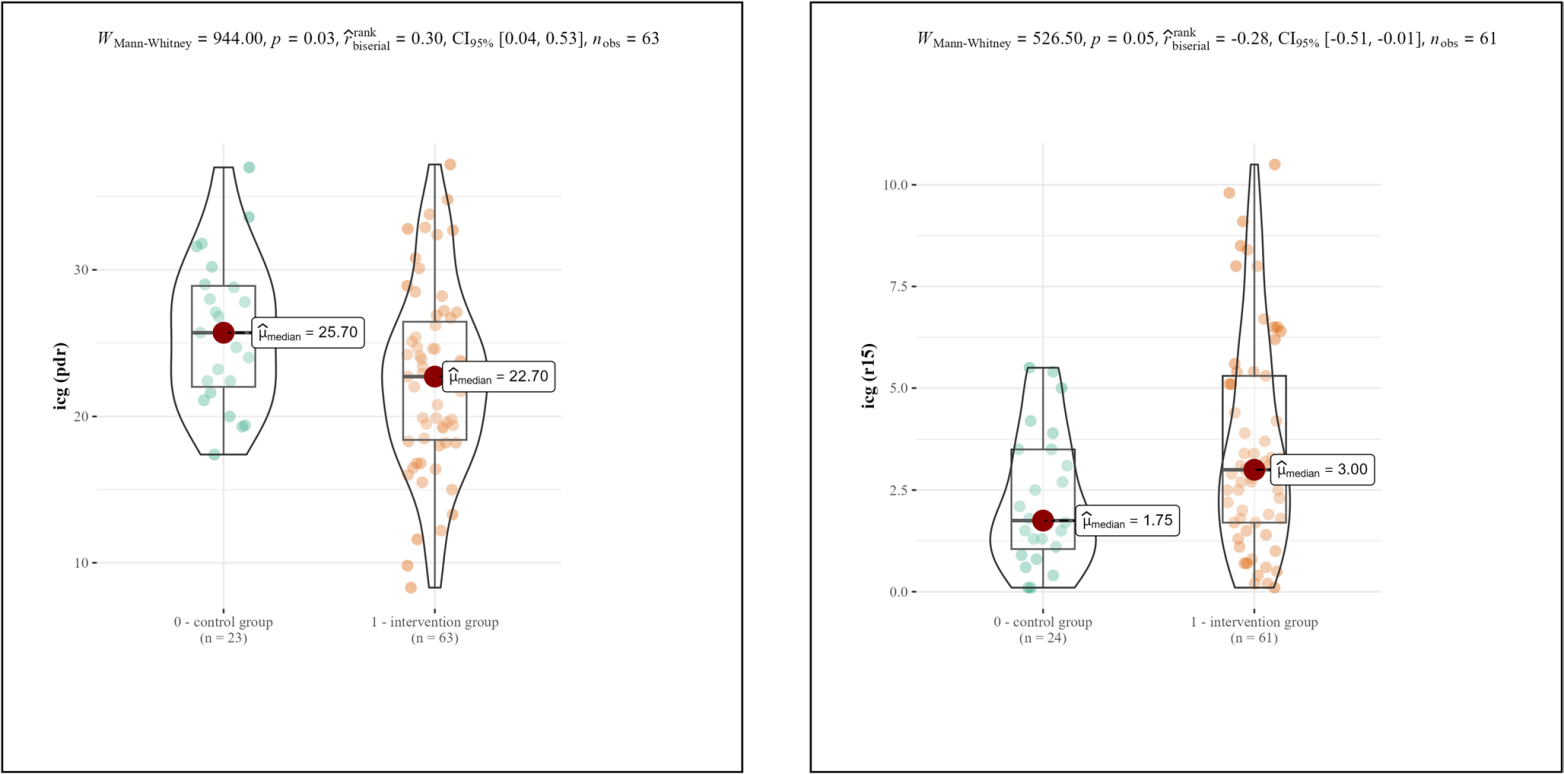
Violin plots comparing ICG-PDR (left) and ICG-R15(right) between the study and control groups. The mean ICG-PDR was higher and ICG-R15 was lower in the control group than in the study group. A Mann-Whitney U test revealed that the difference was statistically significant

A Mann-Whitney U test was conducted to compare ICG clearance between the study and control groups. The difference was statistically significant for both ICG-PDR (*p* = 0.03) and ICG-R15 p(0.05) (Fig. 1).

### Heart Function Monitoring and ICG Clearance

We successfully performed noninvasive heart function measurements at the time of ICG injection in 41 patients. A multiple linear regression analysis was performed to assess the relationship between ICG clearance and cardiovascular parameters (CO, CI, heart rate, systolic blood pressure, and diastolic blood pressure) (Table 2). The model was not statistically significant for ICG-PDR (*p* = 0.1125), but was significant for ICG-R15 overall (*p* = 0.0015). Among the predictors, CO (*p* = 0.002), CI (*p* = 0.008) and diastolic blood pressure (*p* = 0.027) were independently associated with ICG-R15.

**Table 2:** Cardiac parameters and the results of multiple linear regression analysis for ICG-PDR and ICG R15. CO-cardiac output, CI – cardiac index, Psys-systolic blood pressure, Pdias – diastolic blood pressure

			ICG-PDR			ICG-R15		
Predictors	Mean (SD)	CV (%)	Estimates	CI	*p*	Estimates	CI	*p*
(Intercept)			47.75	20.80–74.70	0.001	-1.52	-18.39–15.36	0.858
CO	7.5 (1.6)	21	-0.78	-2.53–0.97	0.377	1.81	0.71–2.91	**0.002**
CI	3.3 (0.7)	21	1.51	-2.52–5.54	0.457	-3.48	-6.00 - -0.96	**0.008**
Heart rate	69.6 (11.7)	17	0.14	-0.07–0.35	0.181	-0.11	-0.24–0.02	0.096
Psys	131.8 (13.2)	10	-0.03	-0.20–0.14	0.706	-0.07	-0.18–0.03	0.169
Pdias	73.4 (5.9)	8	-0.39	-0.80–0.02	0.06	0.29	0.03–0.54	**0.027**
Observations			69			69		
R^2^/R^2^ adjusted			0.129 / 0.060			0.263 / 0.204		

### ICG Clearance Correlation with Steatosis, Lobular Inflammation and Hepatocyte

#### Ballooning

We performed a Kruskal-Wallis one-way ANOVA test to assess the association between ICG clearance (ICG-PDR and ICG-R15) and steatosis, lobular inflammation, and hepatocyte ballooning. After removing the extreme values, no statistically significant association was found (Table 3).

### ICG Clearance Divided by Heart Function Correlation with Steatosis, Lobular Inflammation and Hepatocyte Ballooning

To assess the influence of CO and CI on the correlation between ICG clearance and histological parameters ICG-PDR and ICG-R15 were divided by CO and CI, yielding four new parameters: ICG-PDR/CO, ICG-PDR/CI, ICG-R15/CO, and ICG-R15/CI. We performed a Kruskal-Wallis one-way ANOVA test to assess the association of ICG-PDR/CO, ICG-PDR/CI, ICG-R15/CO, and ICG-R15/CI with steatosis, lobular inflammation, and hepatocyte ballooning. After removing the extreme values, no statistically significant association was found (Table 3).

**Table 3:** Results of Kruskal-Wallis one-way ANOVA test did not show a statistically significant correlation between ICG clearance as expressed by ICG-PDR and ICG-R15, and the histologic evaluation of steatosis, lobular inflammation and hepatocyte ballooning. After correction of both ICG-PDR and ICG-R15 for cardiac output (CO) and cardiac index (CI), there was still no statistically significant correlation

Parameter	Steatosis (*p*)	Lobular inflammation (*p*)	Hepatocyte ballooning (*p*)
PDR	0.27	0.34	0.14
PDR/CO	0.46	0.22	0.91
PDR/CI	0.4	0.9	0.73
R15	0.45	0.13	0.28
R15/CO	0.35	0.2	0.57
R15/CI	0.33	0.23	0.54

### ICG Clearance Correlation with MAS

**Fig. 2:**
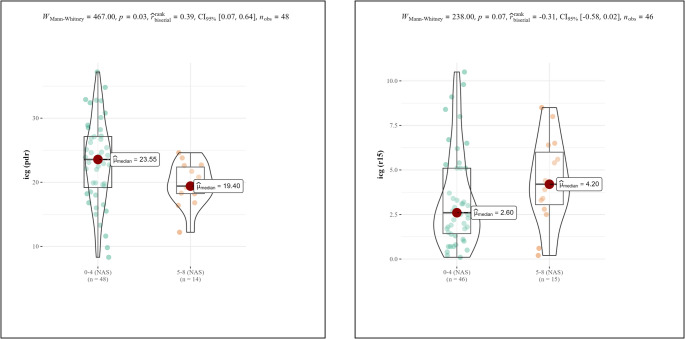
Violin plots comparing ICG-PDR (left) and ICG-R15(right) with MAS. The mean ICG-PDR was higher and ICG-R15 was lower in the MAS > 4 group. A Mann-Whitney U test revealed that the difference was statistically significant for ICG PDR and showed a clear trend for ICG-R15

A Mann-Whitney U test was used to compare ICG clearance with MAS. When comparing low to intermediate risk scores (MAS 0–4) with pathological score (MAS 5–8) the analysis revealed a significant difference between the groups for ICG-PDR (*p* = 0.03) and a clear trend for ICG-R15 (*p* = 0.07) (Fig. 2).

### ICG Clearance Correlation with Fibrosis

Linear regression analysis was performed to assess the relationship between fibrosis and ICG-PDR and ICG-R15. The scatter plot and regression line indicated no clear trend, suggesting a lack of significant association. The Spearman correlation coefficient was close to zero for ICG-PDR and ICG-R15 both, and not statistically significant (Spearman coefficient ≈ 0, *p* = 0.97) (Picture 1).

### ICG Clearance Divided by Heart Function Correlation with Fibrosis

Linear regression analysis was performed to assess the relationship between fibrosis and ICG-PDR and ICG-R15. For ICG-PDR, the scatter plot and regression line indicated a trend, suggesting a significant association for ICG-PDR/CO (Spearman correlation coefficient ≈ 0.38, *p* = 0.02) and ICG-PDR/CI (Spearman correlation coefficient ≈ 0.41, *p* = 0.009).

**Fig. 3:**
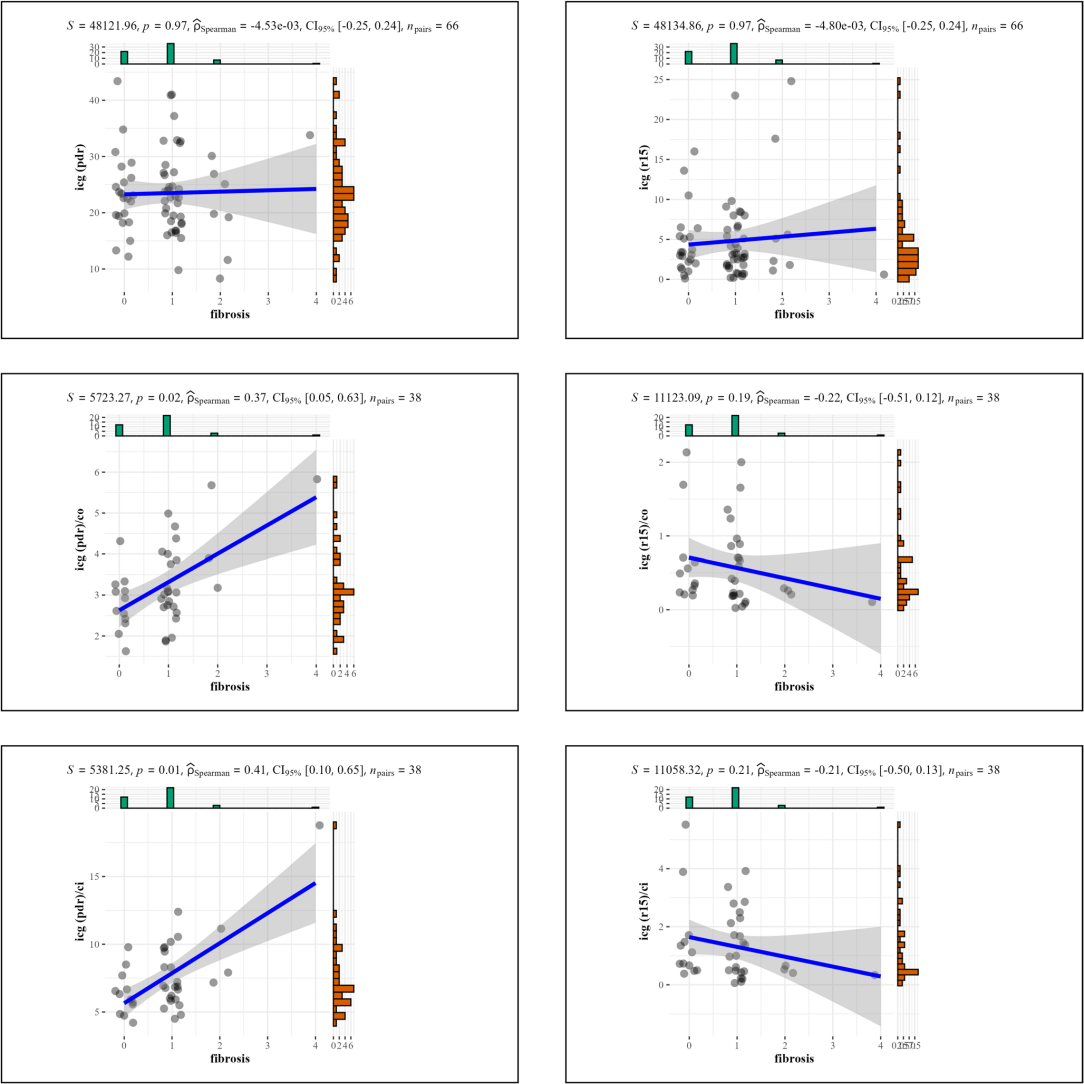
A linear regression analysis assessing the relationship between fibrosis and ICG clearance (ICG-PDR, ICG-R15) and ICG clearance adjusted for CO and CI (ICG-PDR/CO, ICG-PDR/CI, ICG-R15/CO and ICG-R15/CI). The scatter plot and regression line indicated a trend and a significant association for ICG-PDR/CO and ICG-PDR/CI. Although none of the associations reached statistical significance for R15, the decrease in p-values for R15/CO and R15/CI compared to R15 alone suggests a potential trend toward a relationship when adjusting for CO and CI

For ICG-R15, the scatter plot and regression line indicated no observable trend, suggesting no significant association for ICG-R15/CO (Spearman’s correlation coefficient ≈ -0.19, *p* = 0.25) and ICG-R15/CI (Spearman’s correlation coefficient ≈ -0.22, *p* = 0.18) (Fig. 3).

## Discussion

The mortality rate of MASLD increases with an increasing number of components of the metabolic syndrome [23]. Though one of the leading causes of liver transplantation, patients with MASLD are more likely to be denied listing for transplant or to die on the waiting list due to complications of their comorbid disease [24–26].

Lifestyle modifications, including a healthy diet and regular exercise, provide the foundation for the treatment of MASLD [27, 28]. Weight loss required to improve both liver health and other metabolic comorbidities is, however, difficult to achieve, even with structural interventions, and even more difficult to maintain [29, 30]. Pharmaceutical options are few, and no long-term experience is available [31, 32].

Several studies support a substantial benefit of MBS on steatosis, steatohepatitis, fibrosis and a lower incidence of major liver adverse outcomes [1, 2, 33, 34]. However, the lack of randomized control trials precludes the determination of the safety and efficacy of MBS as a formal treatment for MASLD [33]. A major obstacle in MASLD monitoring and subsequent research is the lack of diagnostic capabilities.

Biopsy plays a central role in the diagnosis of MASLD; however, it is limited by availability, invasiveness, and interobserver variability [35–37]. Paired liver biopsies are required to accurately assess the disease response to treatment [33]. However, even paired liver biopsies are limited, as the obtained sample provides information only on a specific portion of a much larger organ. A study of paired liver biopsies obtained at the same time from left and right liver found the agreement was excellent regarding the grade of steatosis (κ = 0,88), moderate for fibrosis (κ = 0.53) but much lower for components of necroinflammation such as lobular inflammation (κ = 0.32) and hepatocyte ballooning (κ = 0.20) [36].

NIT are used to circumvent these limitations and reduce the need for liver biopsy. Several clinical, anthropometric, and biochemical biomarkers have been implicated in MASLD and combined in clinically validated scores, such as FIB-4 and NAFLD fibrosis score [38, 39]. Even with extensive validation, no single test has proven to be superior, and some debate remains regarding the appropriate cutoff points [40, 41]. Imaging modalities such as ultrasound, transient elastography, and magnetic resonance offer insight into the components of MASLD, with no single method being sufficient to meet al.l the needs of clinical practice [42–45].

An interplay of laboratory tests, metabolic workup and imaging modalities is employed to thread the path between MASLD, MASH and fibrosis but the diagnostic accuracy still leaves much to be desired [39, 46, 47].

Liver function monitoring by measuring ICG clearance could be a useful adjunct in the NIT toolbox. The clinical and prognostic value of ICG clearance lies in its ability to provide a dynamic, quantitative measure of global liver function that reflects both perfusion and hepatocellular extraction. Unlike static biochemical markers (e.g., aminotransferases, albumin) or imaging-based tests (e.g., ultrasound elastography, MRI), ICG clearance evaluates real-time hepatic physiology. The method is widely validated and routinely used in clinical practice.

The pathophysiological basis lies in the downregulation of transporter proteins responsible for ICG uptake, including the organic anion transporting polypeptides (OATP) 1B1 and 1B3, and the Na⁺-taurocholate co-transporting polypeptide (NTCP). Experimental models of MASLD and MASH have shown decreased hepatic expression of these transporters, resulting in slower ICG elimination, while in humans reduced OATP expression has been demonstrated in MASH compared with healthy liver tissue [48–54]. These findings were further supported by Danin, who evaluated 26 patients undergoing MBS and compared ICG clearance to the histological evaluation of MASLD, finding a significant correlation between ICG R15 and hepatic steatosis, MAS, and fibrosis [55].

Our study explored the usefulness of ICG clearance in the evaluation of MASLD and partially confirmed these findings.

In our study, ICG clearance distinguished between the groups of patients undergoing bariatric surgery and the healthy control group. We observed a 3.7% difference between ICG PDR (*p* = 0.03) and a 2.3 difference in R15 (*p* = 0.05). While we did not obtain a biopsy in the control group to confirm the presence of MASLD, we believe that the difference in risk factors provides sufficient discrimination. The mean BMI was 43.9 kg/m^2^ in the observed and 25.2 kg/m^2^ in the control group. The risk of MASLD increases with BMI, with a 20% risk increase for each 1-unit increase in BMI [56]. Furthermore, the observed group had a high incidence of comorbidities associated with MASLD. However, it must be stressed that some patients in the control group could still have liver changes consistent with MASLD, which can be found in 18.8% of patients with a BMI < 25 and 32.3% with a BMI < 30 [57]. Although statistically significant, the differences in ICG clearance between MASLD and control patients were modest and should be interpreted as physiological rather than diagnostic. ICG clearance may thus complement, rather than replace, other NITs in the clinical assessment of MASLD.

In contrast to previous studies, we observed no correlation between individual histological features of MASLD and ICG clearance [55]. This might be explained by the remarkable adaptability of the liver in preserving function, especially in the setting of early disease. The compensatory capacity then decreases with the progression of the disease which we believe is reflected in the observed correlation between ICG PDR and a pathological MAS ≥ 5 (*p* = 0.03), where a strong trend was also observed for ICG-R15 (*p* = 0.07). The progression of MASLD to MASH, as observed by the pathologic MAS, seems to correlate with a relevant decline in liver function, as measured by ICG clearance.

Hepatic blood flow is a crucial factor in the interpretation of ICG clearance. The relationship between hepatic clearance (CL) and perfusion can be described as CL = Q x E, where Q represents hepatic blood flow and E the extraction rate. For compounds such as ICG with a high extraction rate, clearance approximates blood flow (CL-ICG ≈ Q) [58]. Consequently, variations in systemic hemodynamics, particularly cardiac output, have a direct impact on measured ICG clearance. As demonstrated by Janssen et al., doubling hepatic perfusion flow markedly improved ICG clearance despite preserved hepatocellular function, confirming its flow-limited nature [12]. Therefore, incorporating cardiac parameters, such as CO and CI, in the interpretation of ICG clearance, may help demask subtle changes of liver extraction otherwise concealed by the variations of systemic circulation. To achieve this, we obtained cardiac function measurements at the time of ICG injection and integrated them into our analysis to minimize the effect of momentary hemodynamic variability on liver perfusion. Indeed, our results show that ICG R15 correlated well with both CO (*p* = 0.002) and CI (0,008). Importantly however, the directions of these associations differed. This likely reflects multicollinearity between CO and CI and the fact that absolute cardiac output does not necessarily translate into proportional hepatic perfusion in obese or MASLD patients, where splanchnic flow distribution may be altered. Therefore, cardiac index, which adjusts for body surface area, may better reflect the functional hepatic blood flow relevant to ICG clearance.

No correlation was observed when comparing unadjusted ICG clearance with the grade of fibrosis. This can be partly attributed to the fact that 89.6% of our patients exhibited no or grade 1 fibrosis, while only one patient exhibited 4 fibrosis (1,3%).

However, when we adjusted the measurements of ICG clearance for CO and CI a trend suggesting association between adjusted ICG clearance and fibrosis grade developed and was statistically significant for ICG PDR/CO (*p* = 0.02) and ICG-PDR/CI (*p* = 0.009).

We believe that liver alterations seen in F1-2 fibrosis, such as early perisinusoidal matrix depositions and sinusoidal capillarization, could be demasked by the inclusion of CO and CI, as the effects of compensatory vasodilatation and altered hepatic arterial buffer response on the measurement are diminished.

To the best of our knowledge, this is the first report of using CO and CI to improve the ability of ICG clearance in identifying early liver disease and is an important milestone in the further improvement of liver function monitoring. This is especially important in identify patients at risk of fibrosis, which is the most important predictor of mortality in MASLD [59].

However, ICG clearance is unlikely to be accurate enough to serve as a standalone diagnostic tool for MASLD. Its main value may lie in broadening the spectrum of noninvasive tests by complementing existing structural and biochemical assessments with a functional dimension. When interpreted together with cardiac function parameters, ICG clearance can help differentiate intrinsic hepatic dysfunction from hemodynamic influences and thereby enhance the diagnostic precision of current noninvasive algorithms for MASLD.

Our study has several limitations that need to be addressed. We used a noninvasive method of cardiac output monitoring using a volume clamp-pulse contour method [60]. This technique requires minimal operator training and allowed the same investigator to perform both cardiac and ICG measurements simultaneously, ensuring precise temporal alignment. It was therefore considered most practical and least invasive for a first study in the ICG-clearance – cardiac function relationship, albeit at the expense of greater accuracy that might be achieved with echocardiography or invasive monitoring. While shown to have good agreement with benchmark invasive measurements in hemodynamically stable conditions, it cannot be used interchangeably with current invasive devices [60, 61]. Further studies utilizing the greater accuracy of invasive methods, such as pulmonary artery catheter bolus thermodilution, could potentially better demask the compensatory changes in liver circulation. The second important weakness was the relatively low number of patients enrolled in the control group, which was a problem of the study design, as it proved difficult to recruit enough individuals within the design parameters. However, the study design allowed the control group to draw some benefit from the ICG injection, as intraoperative cholangiography was possible during later surgery, and the group was still large enough to allow comparison. Finally, the weakness in the general usability of this method needs to be addressed. The requirement for two separate measurements can be cumbersome, time-consuming, and expensive. Ideally, only a single measurement is required. ICG elimination kinetics have been used to measure CO [58]. The development of simultaneous cardiac and liver function monitoring using ICG could provide a more accurate assessment of liver function, especially in the early stages of the disease.

## Conclusion

ICG clearance correlated with MASLD and distinguished individuals at risk from healthy controls. ICG PDR was also able to distinguish patients with a pathologic MASLD Activity Score (MAS ≥ 5). Adjusting the PDR for CO and CI revealed a significant association with liver fibrosis that was not visible using unadjusted measurements.

To the best of our knowledge, this is the first study to show that adjusting ICG clearance for cardiac parameters enhances its correlation with liver disease. Further studies are needed to validate these findings and determine the most accurate and practical method for measuring cardiac parameters in this setting. In addition, longitudinal follow-up studies after MBS are needed to evaluate the role of ICG clearance in the management of MASLD. Overall, ICG clearance could perform a major role in the diagnosis of MASLD, guiding, and monitoring response to treatment.

## Data Availability

The datasets generated and/or analyzed during the current study are not publicly available due to patient privacy restrictions but are available from the corresponding author on reasonable request.

## Declarations

### Declaration of AI and AI-assisted Technologies in the Writing Process

This manuscript was edited for language and clarity using AI-assisted tools (ChatGPT and Paperpal). These tools were employed solely to improve grammar, wording, and readability; they were not used for content generation, data analysis, or substantive contributions to the intellectual content of the work. After using these tools, the authors reviewed and edited the content as needed and take full responsibility for the content of the publication.

### Use of MASLD, MASH, NAFLD and NASH Terminology

MASLD and MASH were coined to more accurately represent the disease, which was, apart from a small difference in the definition, formerly (and in some cases still) known as non-alcoholic fatty liver disease (NAFLD) and non-alcoholic steatohepatitis (NASH). Due to a better understanding of the disease pathophysiology and potentially stigmatizing terminology of “fatty” and “nonalcoholic” the name has been changed to MASLD and MASH, which more accurately represent the disease. Although several of the original studies cited in this manuscript were conducted and published using the NAFLD/NASH terminology, the present manuscript consistently applies the updated MASLD/MASH nomenclature throughout all sections to ensure clarity and terminological consistency.
